# Supplementary material for: Detection of Persistent Organic Pollutants in Omental Adipose Tissue from Patients with Diffuse-Gastric Cancer: A Pilot Study
Source: Cancers (Basel). 2021 Sep 29;13(19):4874. doi: 10.3390/cancers13194874 (PMC8508119; doi:10.3390/cancers13194874)
Supplement: Supplementary file 1 [file cancers-13-04874-s001.zip › cancers-1319057-supplementary.pdf]

cancers

MDPI

Supplementary Materials: Detection of Persistent Organic Pollutants in Omental Adipose Tissue from Patients with Diffuse-Gastric Cancer: A Pilot Study

Martine Perrot-Applanat, Cynthia Pimpie, German Cano-Sancho, Jean Philippe Antignac and Marc Pocard

Table S1. Summary of PCDD/PCDF distributions (median, interquartile range) in the omental fat from individuals with metastatic adenocarcinoma (Diffuse gastric cancer, other tumors, and all tumors groups) (pg /g). The case groups were compared to control group with the Mann-Whitney U test.

Chemicals	Controls (*n* = 8)	Diffuse gastric (*n* = 14)	P-value	Other tumors (*n* = 10)	P-value	All tumors (*n* = 24)	*p*-value
2.3.7.8-TCDD	1.2 (0.6 - 1.2)	1.3 (1.0 - 1.8)	0.19	1.3 (1.0 - 2.3)	0.20	1.3 (1.0 - 1.9)	0.14
1.2.3.7.8-PeCDD	3.6 (2.9 - 4.3)	5.1 (3.4 - 7.3)	0.27	4.8 (3.7 - 7.7)	0.15	4.8 (3.6 - 8.0)	0.15
**1.2.3.4.7.8-HxCDD**	1.1 (1.0 - 1.7)	2.3 (1.4 - 3.8)	**0.04**	2.2 (1.6 - 5.3)	**0.02**	2.3 (1.5 - 4.1)	**0.01**
**1.2.3.6.7.8-HxCDD**	9.7 (7.8 - 12.5)	19.3 (10.4 - 29.3)	**0.04**	16.2 (12.6 - 27.3)	**0.03**	17.4 (11.9 - 30.0)	**0.01**
1.2.3.7.8.9-HxCDD	1.4 (0.9 - 1.8)	1.9 (1.0 - 2.9)	0.27	1.4 (1.1 - 2.9)	0.32	1.7 (1.0 - 3.1)	0.22
**1.2.3.4.6.7.8-HpCDD**	5.8 (5.1 - 7.5)	18.2 (9.3 - 25.3)	**0.03**	15.6 (8.3 - 21.7)	0.06	17.9 (8.8 - 22.9)	**0.02**
**OCDD**	44 (36 - 56)	113 (74.6 - 259)	**0.003**	63 (58 - 95)	0.08	105 (58 - 173)	**0.006**
**Sum PCDDs**	67 (55 - 94)	165 (105 - 319)	**0.005**	102 (87 - 138)	**0.04**	141 (90 - 255)	**0.005**
2.3.7.8-TCDF	0.3 (0.3 - 0.9)	0.3 (0.2 - 0.5)	0.87	0.5 (0.2 - 0.6)	0.97	0.3 (0.2 - 0.5)	0.95
1.2.3.7.8-PeCDF	0.2 (0.1 - 0.4)	0.1 (0.1 - 0.2)	0.66	0.3 (0.2 - 0.4)	0.27	0.2 (0.1 - 0.3)	0.81
2.3.4.7.8-PeCDF	9.2 (6.9 - 12.0)	15.0 (8.2 - 20.4)	0.13	12.4 (10.2 - 19.2)	0.08	13.9 (8.6 - 20.2)	0.06
**1.2.3.4.7.8-HxCDF**	1.7 (1.5 - 2.3)	2.9 (2.1 - 4.4)	0.11	2.6 (2.2 - 3.5)	**0.04**	2.7 (2.1 - 4.0)	**0.04**
**1.2.3.6.7.8-HxCDF**	2.0 (1.6 - 2.8)	3.1 (2.1 - 5.1)	0.15	3.2 (2.5 - 4.7)	**0.04**	3.1 (2.2 - 5.1)	**0.05**
1.2.3.7.8.9-HxCDF	0.1 (0.1 - 0.2)	0.1 (0.1 - 0.2)	0.92	0.1 (0.1 - 0.2)	0.97	0.1 (0.1 - 0.2)	0.92
2.3.4.6.7.8- HxCDF	0.7 (0.5 - 0.9)	1.1 (0.6 - 1.7)	0.11	1.0 (0.6 - 1.5)	0.12	1.0 (0.6 - 1.6)	0.07
1.2.3.4.6.7.8-HpCDF	1.1 (1.0 - 1.2)	1.4 (0.9 - 3.0)	0.24	1.4 (1.0 - 1.8)	0.17	1.4 (0.9 - 2.2)	0.15
1.2.3.4.7.8.9-HpCDF	0.1 (0.1 - 0.1)	0.2 (0.1 - 0.3)	0.15	0.1 (0.1 - 0.2)	0.20	0.2 (0.1 - 0.2)	0.11
OCDF	0.2 (0.2 - 0.3)	0.3 (0.3 - 0.5)	0.05	0.3 (0.2 - 0.4)	0.24	0.3 (0.2 - 0.5)	*0.06*
Sum PCDFs	17.7 (12.9 - 21.0)	23.4 (15.8 - 34.2)	0.11	21.4 (18.5 - 35.8)	0.10	22.5 (17.0 - 35.6)	*0.06*
**WHO-TEQ PCDD/F (TEF2005)**	9.5 (7.2 - 11.7)	14.1 (9.0 - 20.4)	0.15	12.6 (10.3 - 19.6)	0.07	13.2 (9.4 - 21.3)	*0.06*

In bold, significance represented by p<0.05 for comparison with control.

Table S2. Summary of the distribution (median, interquartile range) of PCBs in the omental fat from individuals with metastatic adenocarcinoma (Diffuse gastric cancer, other tumors, and all tumors groups) (pg/g). The case groups were compared to control group with the Mann-Whitney U test.

	Controls (n=8)	Diffuse gastric (n=14)	P-value	Other tumors (n=10)	P-value	All tumors (n=24)	P-value
PCB 77	2.3 (1.6 - 4.2)	1.9 (1.5 - 3.3)	0.87	3.1 (2.3 - 4.3)	0.57	2.4 (0.8-3.3)	0.87
PCB 81	1.2 (0.8 - 3.3)	1.0 (0.7 - 2.3)	0.57	2.4 (1.6 - 3.8)	0.17	1.6 (0.4-11.6)	0.78
PCB 126	45 (31 -61)	42 (24 - 92)	0.82	79 (57 - 98)	*0.05*	68 (11-499)	0.27
PCB 169	78 (41 -111)	117 (74 - 143)	0.27	111 (90 - 193)	0.10	117 (27-255)	0.12
Sum Copl. PCBs	129 (101 -180)	151 (121 - 248)	0.44	189 (160 - 283)	*0.05*	166 (46-789)	0.14
**PCB 105**	3217 (1604-4005)	3541.5 (1928 - 5540)	0.53	6300 (3597 -12064)	**0.03**	4468 (886-30623)	0.15
PCB 114	1085 (5051-2353)	1559 (1235 - 2010)	0.62	2390 (1318 - 3719)	0.15	1735 (224-9730)	0.29
PCB 118	15516 (7550-17555)	17402 (9629 - 24966)	0.40	30968 (17172 - 56890)	0.055	21496 (3676-176519)	0.13
**PCB 123**	133 (6 -155)	139 (65 - 284)	0.57	263 (195 - 504)	**0.02**	199 (19-2085)	0.12
PCB 156	16529 (5050 - 24869)	24763 (15818 - 32199)	0.37	24793 (17099 - 41779)	0.20	24763 (4436-67065)	0.22

	Controls (n=8)	Diffuse gastric (n=14)	P-value	Other tumors (n=10)	P-value	All tumors (n=24)	P-value
PCB 157	2745 (1115-4501)	4584 (3252 - 5519)	0.21	5136 (3952 - 7858)	*0.07*	4788 (995-13614)	*0.08*
PCB 167	2336 (1345-3962)	3360.5 (2843 - 5490)	0.37	4580 (3249 - 6312)	0.08	3933 (785-20053)	0.15
PCB 189	2458 (684 -3210)	4088 (2275 - 5095)	0.095	2950 (2742 - 5980)	0.12	3796 (698-8718)	*0.06*
Sum Non Cop. PCBs	39659 (26274-66540)	54709 (48975 - 70840)	0.27	78909 (50996 - 146650)	*0.05*	64345(12124-327362)	0.09
WHO-TEQ dl-PCB (TEF 2005)	9.4 (6.0 - 10.7)	9.5 (7.3 - 16.2)	0.53	13.3 (11.8 - 16.6)	**0.02**	11.4 (2.5-67.2)	0.12
TOTAL-TEQ (TEF 2005)	19.6 (12.5 - 21.3)	22.6 (18.3 - 36.8)	0.15	24.7 (23.0 - 35.9)	**0.03**	23,8 (7.55-118)	**0.04**
PCB 28	1.3 (1.1 - 1.9)	1.3 (0.8 - 1.7)	0.71	1.5 (0.9 - 2.9)	0.83	1.4 (0.4-16.7)	0.90
PCB 52	0.5 (0.2 - 0.6)	0.2 (0.1 - 0.3)	*0.035*	0.2 (0.2 - 0.6)	0.27	0.22 (0.1-2.4)	0.06
PCB 101	0.7 (0.4 - 1.4)	0.5 (0.2 - 0.7)	0.21	0.6 (0.5 - 1.1)	0.83	0.5 (0.1-8.9)	0.35
**PCB 138**	35 (31.7 - 92.6)	79 (53 - 113.)	0.19	92 (67 - 137.)	**0.04**	80 (18-262)	0.06
PCB 153	97 (66 - 198)	194 (137 - 261)	0.21	238 (147 - 255)	0.10	196 (52-566)	0.10
PCB 180	141 (51 - 180)	233 (138 - 316)	0.11	190 (178 - 315)	*0.05*	226 (50-512)	0.05
**Sum 6 ndl-PCB**	288 (157 - 448)	495 (346 - 666)	0.19	574 (387 - 758)	**0.04**	522 (128-1344)	0.06

In bold, significance represented by p<0.05 for comparison with control.

Table S3. Summary of the distribution (median, interquartile range) of polybromodiphenylethers (PBDE) and polybromobiphenyls (PBB) in the omental fat from individuals with metastatic diffuse adenocarcinoma (Diffuse gastric cancer, other tumors, and all tumors groups),(ng/g). The case groups (ADCI or other tumors) were compared to control group with the Mann-Whitney U test.

	Controls (n = 8)	Diffuse gastric (n = 14)	p-value	Other Tumors (n = 10)	p-value	all tumors (n = 24)	p value
PBDE 28	0.016 (0.014 - 0.028)	0.016 (0.012 - 0.020)	0.40	0.009 (0.005 - 0.028)	0.24	0.013 (0.006 - 0.022)	0.25
PBDE 47	0.151 (0.106 - 0.245)	0.165 (0.048 - 0.330)	0.82	0.075 (0.033 - 0.212)	0.27	0.125 (0.040 - 0.250)	0.48
PBDE 99	0.038 (0.026 - 0.065)	0.047 (0.012 - 0.066)	0.97	0.022 (0.011 - 0.064)	0.46	0.043 (0.010 - 0.070)	0.75
PBDE 100	0.074 (0.053 - 0.097)	0.076 (0.052 - 0.138)	0.71	0.067 (0.051 - 0.083)	0.70	0.067 (0.051 - 0.128)	0.98
PBDE 153	0.847 (0.636 - 2.613)	2.141 (1.682 - 2.598)	0.09	1.831 (1.638 - 2.070)	0.32	1.974 (1.652 - 2.399)	0.11
PBDE 154	0.015 (0.010 - 0.020)	0.019 (0.013 - 0.031)	0.40	0.021 (0.016 - 0.028)	0.32	0.019 (0.014 - 0.030)	0.29
PBDE 183	0.178 (0.068 - 0.321)	0.219 (0.156 - 0.389)	0.37	0.289 (0.228 - 0.366)	0.10	0.244 (0.209 - 0.382)	0.16
**PBDE 209**	1.860 (1.260 - 2.786)	5.014 (3.112 - 9.353)	**0.005**	2.755 (2.336 - 6.320)	0.10	4.844 (2.514 - 9.348)	**0.009**
Sum 7 i PBDE	1.346 (0.963 - 3.372)	2.627 (2.221 - 3.591)	0.09	2.392 (2.213 - 2.976)	0.27	2.503 (2.203 - 3.042)	0.10
**PBB 153**	0.506 (0.340 - 0.574)	1.069 (0.523 - 1.489)	**0.042**	0.766 (0.673 - 1.163)	**0.006**	0.915 (0.645 - 1.460)	**0.008**

In bold, significance represented by p<0.05 for comparison with control.
